# Top‐Down Proteomics Study of Aging and Sexual Dimorphism of Zebrafish Brains

**DOI:** 10.1002/jms.70009

**Published:** 2025-12-30

**Authors:** Mehrdad Falamarzi Askarani, William Poulos, Maryam Rahimzadeh Dashtaki, Seyed Amirhossein Sadeghi, Jose B. Cibelli, Fei Fang, Liangliang Sun

**Affiliations:** ^1^ Department of Chemistry Michigan State University East Lansing Michigan USA; ^2^ Department of Animal Science Michigan State University East Lansing Michigan USA; ^3^ Department of Large Animal Clinical Sciences Michigan State University East Lansing Michigan USA

**Keywords:** aging, CZE‐MS/MS, proteoform, sexual dimorphismtop‐down proteomicszebrafish brain

## Abstract

Aging significantly impacts brain function and increases susceptibility to neurodegenerative diseases, with notable sex dimorphism observed in aging‐related dementia, such as Alzheimer's disease. To better understand the molecular mechanisms of aging and dementia, it is essential to globally and accurately characterize biomolecules (e.g., proteins) in the brain as a function of age and sex. Here, we present one of the first studies of aging and sexual dimorphism in brains using the zebrafish ( *Danio rerio* ) model, employing mass spectrometry (MS)‐based quantitative top‐down proteomics (TDP). We analyzed proteoforms in male and female zebrafish brains across three ages (6, 16, and 24 months) using capillary zone electrophoresis‐tandem MS (CZE‐MS/MS). We revealed significant sex‐ and age‐related differences in the abundance of key proteoforms, including those associated with neurofilament assembly, dopaminergic neuron differentiation, and synaptic vesicle priming. Notably, we identified a truncated proteoform of AP2B1, a subunit of the AP2 adaptor complex closely associated with clathrin‐mediated endocytosis and cellular aging, in female zebrafish aged 24 months exclusively, suggesting potential age‐ and sex‐specific roles in brain aging. Additionally, significant changes were observed in proteoforms involved in energy metabolism, structural maintenance, and neurotransmitter release, providing a new opportunity for a better understanding of the molecular mechanisms of brain aging and sexual dimorphism. These findings highlight the effectiveness of CZE‐MS/MS in TDP for identifying and quantifying proteoforms, offering a deep view of sex‐specific proteoform dynamics during brain aging.

## 1 Introduction

Aging is a complex biological process characterized by a progressive decline in physiological function, ultimately leading to increased vulnerability to various diseases [1]. In particular, brain aging is characterized by a slow decline in cognitive abilities, such as memory, attention, learning, and decision‐making, all of which have a major influence on life quality [2]. Dementia, e.g., Alzheimer's disease (ad), is closely related to brain aging, and sex dimorphism in dementia is widespread [3]. Sex dimorphism of the brain is influenced by both genetic factors encoded in sex chromosomes and hormones. To achieve a better understanding of the molecular mechanisms underlying brain aging and related dementia, we need to pursue global and accurate characterization of biomolecules in the brain as a function of age and sex.

Proteins modulate almost all the biological processes in cells. Proteomics has been widely used in studying the brain proteome, sex dimorphism, brain aging, and related dementia [4, 5, 6, 7, 8, 9, 10]. Those studies have significantly advanced our understanding of brain aging and related diseases. All the studies have employed the mass spectrometry (MS)–based bottom‐up proteomics (BUP) approach, which identifies and quantifies proteins and post‐translational modifications (PTMs) by measuring the peptides produced by enzymatic digestion of proteins with extremely high sensitivity. However, BUP cannot offer the characterization of proteoforms representing various forms of protein molecules derived from the same gene due to RNA alternative splicing and protein PTMs because of the loss of intact proteoform information during the enzymatic digestion step [11, 12, 13, 14, 15]. Proteoforms play critical roles in modulating disease progression [16, 17, 18, 19, 20]. A comprehensive characterization of proteoforms in the male and female brains as a function of age is crucial for a better understanding of brain aging and sex dimorphism.

MS‐based top‐down proteomics (TDP) is ideal for measuring proteoforms by directly separating and detecting intact proteoforms, rather than peptides [17, 21, 22, 23, 24]. It has been widely used in studying proteoforms involved in various diseases, e.g., cancer, cardiovascular diseases, infectious diseases, and neurodegenerative diseases [25, 26, 27, 28, 29]. Several studies have used MS‐based TDP to study brain proteoforms [30, 31, 32]. The Kelleher group discovered substantial differences in the proteoform profiles of brain tissues from four healthy mouse strains [32]. The Petyuk group conducted a comprehensive TDP study of human ad brain tissue using reversed‐phase liquid chromatography (RPLC)–high‐field asymmetric waveform ion mobility spectrometry (FAIMS)–MS/MS, identifying over 5000 proteoforms [31]. Our group performed quantitative TDP of zebrafish male and female brains at 11 months to determine the sexual dimorphism of the brain at the proteoform level [30]. We revealed a substantial difference in the proteoform profile between male and female brains. In this study, we extended our research to study the proteoforms in male and female zebrafish brains as a function of age (6, 16, and 24 months) to better understand brain aging and sexual dimorphism.

## 2 Experimental Section

### 2.1 Materials and Reagents

Ammonium bicarbonate (ABC), dithiothreitol (DTT), and Amicon Ultra (0.5 mL, 30 kDa molecular weight cut‐off) centrifugal filter units were ordered from Sigma‐Aldrich (St. Louis, MO). LC/MS grade water, LC‐grade acetic acid (AA), formic acid, and methanol fused silica capillaries (50 μm i.d., 360 μm o.d., Polymicro Technologies) were purchased from Fisher Scientific (Pittsburgh, PA). Acrylamide was obtained from Acros Organics (Fair Lawn, NJ). Complete, mini protease inhibitor cocktail and PhosSTOP (EASYpacks) were purchased from Roche (Indianapolis, IN).

### 2.2 Sample Preparations

Zebrafish brain samples from both males and females were collected at three different ages: 6, 16, and 24 months, representing young, middle‐aged, and old, respectively. Three zebrafish per age group were used in this study. All protocols involving zebrafish were carried out in compliance with relevant laws, guidelines, and regulations set forth by the Institutional Animal Care and Use Committee (IACUC) at Michigan State University.

To remove the blood, the collected brains were gently washed three times with Dulbecco's phosphate‐buffered saline (Fisher Scientific, Pittsburgh, PA). The tubes containing tissue samples were snap frozen in liquid nitrogen and stored at −80°C until analysis. To extract the proteins, male and female zebrafish brains were lysed using 8 M urea and 100 mM ABC (pH 8.0) containing protease inhibitors and phosphatase inhibitors. The brain tissues were homogenized using a Fisher Scientific Homogenizer 150 and sonicated on ice with a VWR Scientific Branson Sonifier 250 to facilitate protein extraction. After centrifugation at 18 000 × g for 10 min at 15°C to remove lipids and cellular debris, the protein‐containing supernatant was collected. The protein concentration in the supernatant was determined using a bicinchoninic acid (BCA) assay (Fisher Scientific, Pittsburgh, PA). Before MS analysis, the zebrafish brain proteins were reduced with 1 M DTT at 37°C for 30 min.

In this study, an Amicon Ultra Centrifugal Filter with a molecular weight cut‐off (MWCO) of 30 kDa was utilized to remove urea from protein samples through buffer exchange. Initially, the filter was prepared with 100 μL of 100 mM ABC (pH 8.0) and centrifuged at 14 000 g for 15 min. Following this, each protein sample was equally transferred to three centrifugal filters, with around 130 μg of protein in each filter. Subsequently, a 50 mM ABC solution was added to each filter to make a final volume of 200 μL. Further centrifugation at 14 000 g for 20 min at 15°C was carried out. Two additional rounds of buffer exchange were performed by adding 50 mM ABC to each filter to reach a final volume of 200 μL. All steps were conducted at 4°C for 20 min to ensure thorough removal of urea. The centrifugal filter was then gently vortexed for 20 min and thoroughly pipetted. Afterward, the filter assembly was moved to a new Eppendorf tube for sample collection. By inverting the filter assembly and centrifuging it at 3000 g for 3 min, the protein sample (20–30 μL protein solution) was collected. The resulting sample was prepared for capillary zone electrophoresis (CZE)‐electrospray ionization (ESI)‐MS/MS analysis. The total protein concentration was determined using a BCA kit following the manufacturer's protocol and stored at 4°C. The protein solution was then analyzed by CZE‐MS/MS on the same day.

### 2.3 CZE‐ESI‐MS/MS Analysis

The system configuration for CZE‐MS/MS consisted of a ceSI 8000 Plus ce system from Beckman Coulter and an Orbitrap Exploris 480 mass spectrometer from ThermoFisher Scientific. This was achieved by utilizing an electrokinetically pumped sheath‐flow CE‐MS nanospray interface [33, 34]. A fused silica capillary (50 μm i.d./360 μm o.d., 1 m in length) was coated with linear polyacrylamide (LPA) [35]. Hydrofluoric acid was used to etch the capillary, reducing the outer diameter of one end to 70–80 μm [36]. A 100 nanoliter of each sample (~1.3 mg/mL protein concentration) was injected into the separation capillary for analysis by applying a 5‐psi pressure for 19 s, following the principles of Poiseuille's law. The CZE separation was carried out by applying a 30‐kV voltage at the sample injection end for 80 min. A 2‐kV voltage was applied at the ce‐MS interface for ESI. A pressure of 50 psi was applied to flush the capillary between runs for 10 min using the background electrolyte (BGE). An electrospray ionization (ESI) emitter was created from a glass capillary (0.75 mm i.d./1.0 mm o.d., 10 cm in length) with a Sutter P‐1000 flaming/brown micropipette puller. The emitter orifice ranged from 20 to 40 μm. The BGE of CZE was 5% (v/v) acetic acid (pH 2.4). The sheath buffer for ESI was 0.2% (v/v) formic acid and 10% (v/v) methanol.

An Orbitrap Exploris 480 mass spectrometer was used for all experiments. A data‐dependent acquisition (DDA) mode was used for data acquisition. In this condition, a mass resolution of 120 000 (at m/z 200) with a single microscan and a scan range of 600–2000 m/z was used. The normalized AGC target value of 300% and the auto maximum injection time were employed for the parent ion scan (MS1). The precursor ions were isolated using a 2‐m/z window and then fragmented using higher energy collisional dissociation (HCD) with a normalized collision energy (NCE) of 25%. Only precursor ions with an intensity greater than 10 000 and a charge state between 5 and 60 were chosen for fragmentation. MS/MS spectra were acquired at a resolution of 60 000 (at m/z 200) with three microscans, a normalized AGC target of 100%, and the number of dependent scans was 12. Dynamic exclusion was activated for 30 s, using a mass tolerance of 10 ppm, and the “Exclude isotopes” feature was activated.

### 2.4 Data Analysis

The data were analyzed using Xcalibur software (Thermo Fisher Scientific Version 4.5) to obtain the intensity and migration time of proteoforms. The electropherograms were exported using Xcalibur and processed using Inkscape (Version 1.3.2) to obtain the final figures.

The process of identifying and quantifying proteoforms in zebrafish brains from the MS RAW files was carried out using the TopPIC (Top‐down mass spectrometry–based Proteoform Identification and Characterization) pipeline [37]. In the initial stage, the RAW files were converted into mzML files using the Msconvert tool [38]. The process of converting precursor and fragment isotope clusters into monoisotopic masses and proteoform features was carried out using TopFD (Top‐down mass spectrometry Feature Detection, Version 1.7.0) [39]. The mass spectra and proteoform feature information were saved in msalign and text files, respectively. The database search was conducted via TopPIC (Version 1.7.0). The maximum number of unexpected mass shifts was one. The mass error tolerances for precursors and fragments were 10 ppm. There was a maximum mass shift of 500 Da for unknown mass shifts. To determine the false discovery rates (FDRs) of proteoform identifications, the target‐decoy strategy was employed. Proteoform identifications were then filtered based on a 1% FDR at the proteoform‐spectrum‐match (PrSM) level and a 5% FDR at the proteoform level [40, 41]. Proteoforms that passed the FDR threshold were considered confidently identified. The PrSM cluster error tolerance was set to the default value of 1.2 Da to reduce proteoform redundancy. The lists of identified proteoforms from all CZE‐MS/MS runs are shown in Supporting Information II. The TopDiff (Top‐down mass spectrometry–based identification of Differentially expressed proteoforms, Version 1.7.4) software was used to perform label‐free quantification of identified proteoforms by CZE‐MS/MS using default settings [42]. For quantification analysis between male and female samples across three age groups (6, 16, and 24 months), TopDiff output files were imported into the Perseus software platform for comprehensive statistical evaluation [43]. Data preprocessing in Perseus applied the following filtering criteria to define the quantified proteoforms when we compared any two age groups: Proteoforms need to have valid intensity in at least two out of the three technical replicates in at least one age group. For the missing proteoform intensity values in some replicates, we addressed the issue through imputation using a normal distribution–based approach in Perseus. Subsequently, the quantified proteoforms were used for Student's *t*‐test analysis within Perseus to determine the proteoforms exhibiting statistically significant differences in abundance between any two age groups.

## 3 Results and Discussion

### 3.1 Quantitative TDP of Zebrafish Brains Revealed Substantial Proteoform Changes in the Brain During the Aging Process

Zebrafish is an attractive model organism for studying the molecular mechanisms of brain aging because of its several valuable features. First, zebrafish have a short lifespan (~3 years), which facilitates research on aging. For example, 6, 16, and 24 months old in zebrafish correspond to young, mid, and old ages [44]. Second, zebrafish and human brains share similar overall structures, cell types, and neurotransmitter systems, making zebrafish an excellent model for studying human brain function, aging, and diseases [45]. Quantitative MS‐based BUP has been used to investigate brain aging processes in zebrafish [46, 47, 48]. However, to our best knowledge, no quantitative MS‐based TDP studies have been conducted to study brain aging in a proteoform‐specific manner using the zebrafish model.

**FIGURE 1:**
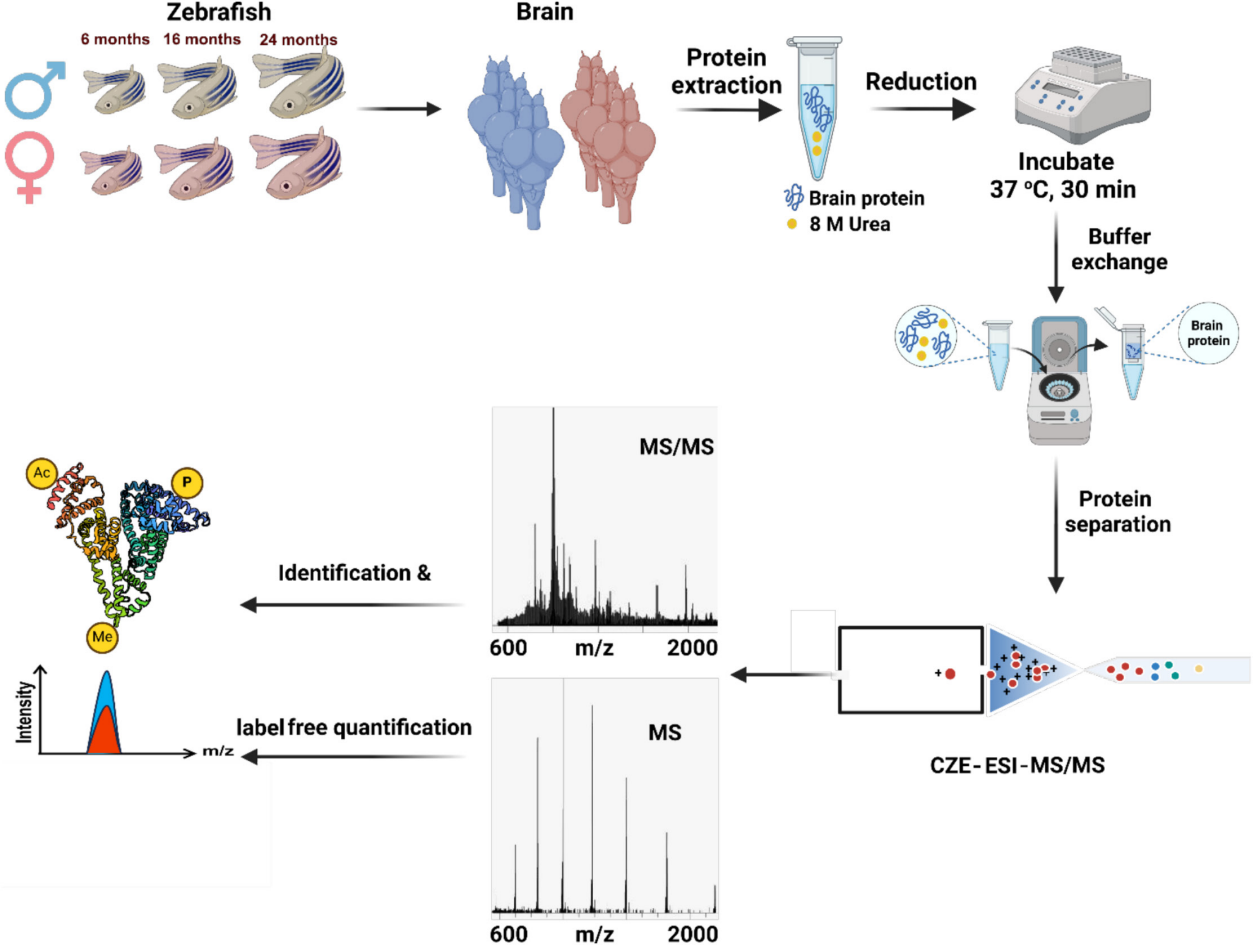
Schematic of the experimental design. The figure is created using BioRender and is used here with permission.

In this study, we utilized CZE‐MS/MS‐based TDP [49, 50] to quantitatively compare the proteoform abundance change as a function of age [6 (young), 16 (middle), and 24 (old) months]. We studied male and female zebrafish brains separately (Figure 1). Three mixed brains at different ages (6, 16, and 24 months) were lysed using homogenization and sonication. The proteoform extract was analyzed by the dynamic pH junction‐based CZE‐MS/MS [51]. Following a straightforward buffer exchange using a centrifugal filter device with a 30‐kDa molecular weight cutoff (MWCO), our lab has employed dynamic pH junction‐based CZE‐MS/MS for TDP of various complex biological samples, e.g., bacteria [51, 52, 53], yeast [54, 55, 56], human cells [22, 57], tissues [58, 59], human plasma [60], plants [61], and histones [62, 63, 64]. In this study, six zebrafish brain samples (three males and three females of different ages) were obtained. Each sample was processed by buffer exchange in technical triplicate. In total, 18 zebrafish brain samples were analyzed by CZE‐MS/MS.

CZE‐MS/MS analysis of the 16‐month‐old male zebrafish brain sample produced consistent proteoform intensity across technical triplicates, evidenced by the high Pearson's correlation coefficients (Figure S1). The data indicate the quantitative reproducibility of the CZE‐MS/MS technique and its suitability for the quantitative TDP measurement of zebrafish brain samples.

**FIGURE 2:**
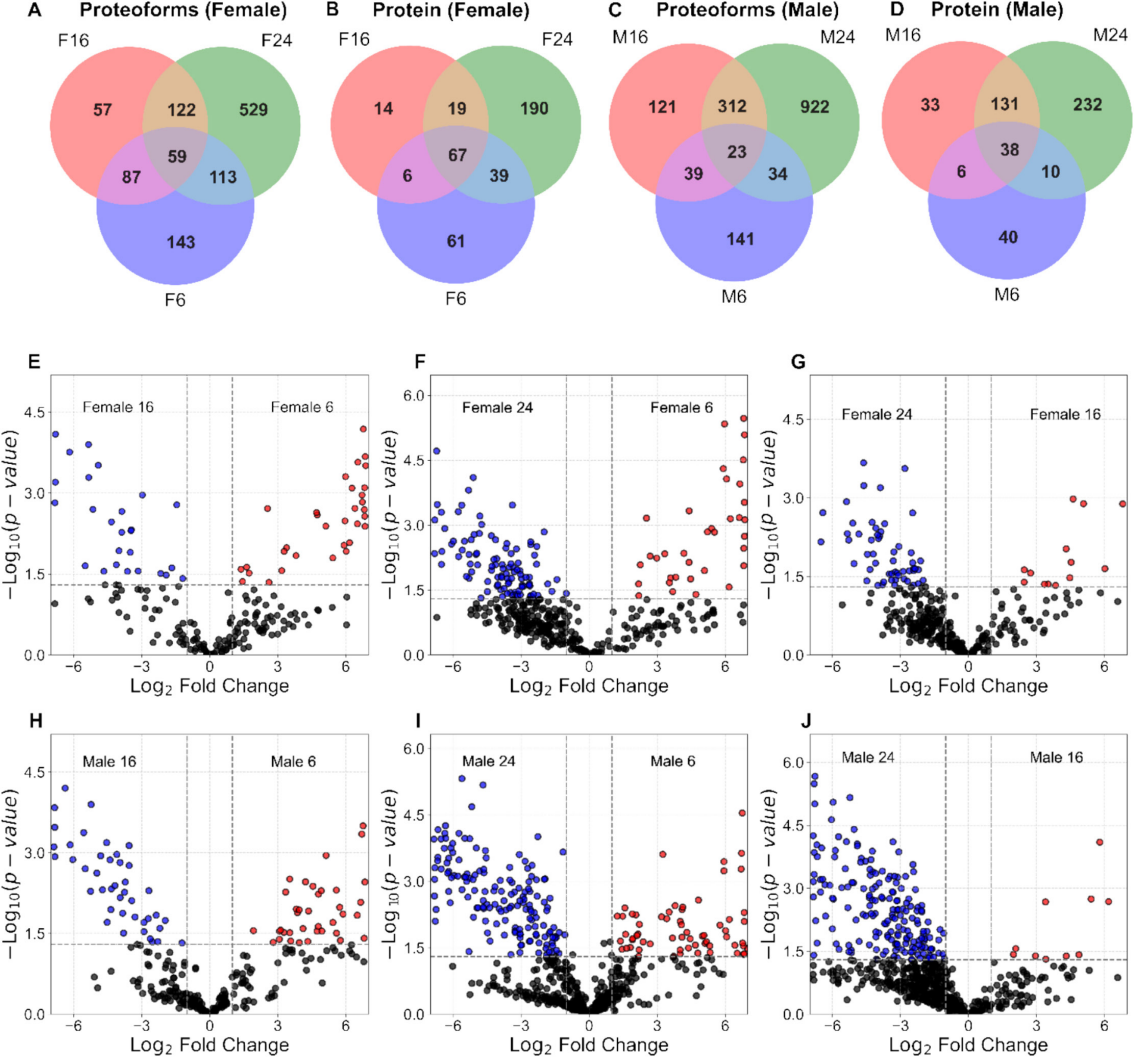
TDP data of zebrafish male and female brains, focusing on different ages. Venn diagrams of identified proteoforms and proteins across the three ages (6, 16, and 24 months): (A) female proteoforms, (B) female proteins, (C) male proteoforms, and (D) male proteins. Volcano plots of quantified proteoforms from female (E, F, and G) and male (H, I, and J) brains between any two ages. Volcano plots display ‐log(*p*‐value) versus the log2 fold‐change for quantified proteoforms in various comparisons: (E) female6/female16, (F) female6/female24, (G) female16/female24, (H) male6/male16, (I) male16/male24, and (J) male6/male24. The vertical and horizontal dotted lines show the cut‐off of log2 fold‐change (± 1) and of *p* = 0.05, respectively. The volcano plot was generated using Python software (Version 3.11.4) [65].

This study investigates age‐related changes in the proteome of zebrafish brains across three life stages (6, 16, and 24 months) using label‐free quantitative proteomics with CZE‐MS/MS. A total of 1106 proteoforms and 396 proteins were identified in females, while 1707 proteoforms and 490 proteins were identified in males, with a proteoform‐level FDR of 5%. The Venn diagrams illustrate the overlap and age‐specific distribution of proteoforms and proteins across life stages and sex (Figure 2A–D). In females, 59 proteoforms were shared among all three age groups, while 143, 57, and 529 proteoforms were unique to 6, 16, and 24 months, respectively (Figure 2A). Notably, our findings revealed that in female samples aged 24 months, a truncated proteoform of AP2B1 was identified with a mass shift of 250.125 Da, suggesting potential age‐specific roles (Figure S2). The AP2 adaptor complex, particularly its alpha subunit AP2A1, has recently been shown to play a central role in clathrin‐mediated endocytosis and cellular aging. AP2A1 is highly expressed in senescent cells, and its suppression in older cells reverses the aging process. In contrast, its overexpression in young cells accelerates senescence, making it a promising biomarker and therapeutic target for age‐related diseases [66]. The AP2 complex, including its beta subunit AP2B1, is also involved in neurodegenerative processes, such as its interaction with amyloid precursor protein (APP) in Alzheimer's disease, where it helps regulate APP trafficking and prevent neuronal dysfunction [67]. These findings highlight the multifaceted functions of AP2 complex proteins in endocytosis, aging, and neurodegeneration, positioning them as key targets for further research and therapeutic development.

Similarly, in males, 23 proteoforms were shared, with 141, 121, and 922 unique to each respective age (Figure 2C). Protein‐level analysis showed 67 and 38 proteins shared across all ages in females and males, respectively, while age‐specific proteins were predominantly identified at 24 months (Figure 2B,D). These findings underscore the dynamic nature of the aging brain proteome and highlight 24 months as a critical time point for molecular changes.

We determined the differentially expressed proteoforms in zebrafish brains between any two ages studied (6, 16, and 24 months) based on label‐free quantification. For female brains, we quantified 197, 426, and 366 proteoforms of 106, 200, and 172 proteins when we compared 6 and 16 months, 6 and 24 months, and 16 and 24 months, respectively. Among the quantified proteoforms, 59, 142, and 69 proteoforms of 36, 82, and 44 proteins showed statistically significant differences in abundance between the ages compared, i.e., female 6‐ vs. 16‐month, 6‐ vs. 24‐month, and 16‐ vs. 24‐month age groups, respectively (Figure 2E–G). For male brains, 281, 655, and 651 proteoforms of 140, 272, and 259 proteins were quantified when we compared the 6‐ and 16‐month, 6‐ and 24‐month, and 16‐ and 24‐month age groups, respectively. Among these, 81, 226, and 193 proteoforms of 57, 126, and 126 proteins exhibited statistically significant differences in abundance between the age groups compared, i.e., female 6‐ vs. 16‐month, 6‐ vs. 24‐month, and 16‐ vs. 24‐month time points, respectively (Figure 2H–J). The differentially expressed proteoforms of all the comparisons are listed in Supporting Information II. The results demonstrate that as the zebrafish brain ages, its proteoform profile undergoes substantial changes for both males and females.

We investigated the changes of PTMs (i.e., phosphorylation) during the aging process of the zebrafish brain. Phosphorylation is a well‐established regulator of cellular signaling, gene expression, neuronal differentiation, and brain aging [68, 69]. We selected four phosphorylated proteoforms that exhibit differential expressions in the brain during aging as examples (Figure S3), including one phosphorylated proteoform of Hmgn6 (High Mobility Group Nucleosome‐Binding Domain 6), Calm1a (Calmodulin), Dpysl3 (Dihydropyrimidinase‐Related Protein 3), and Nfma (Neurofilament Medium Chain a). Dpysl3 is particularly notable because of its role in cytoskeletal organization, a process highly vulnerable to age‐associated regulation. Altered phosphorylation of Dpysl3 could impact neuronal dysfunction and cognitive decline during aging [70]. The results here indicate substantial changes in phosphorylated proteoforms during the aging process of the zebrafish brain.

**FIGURE 3:**
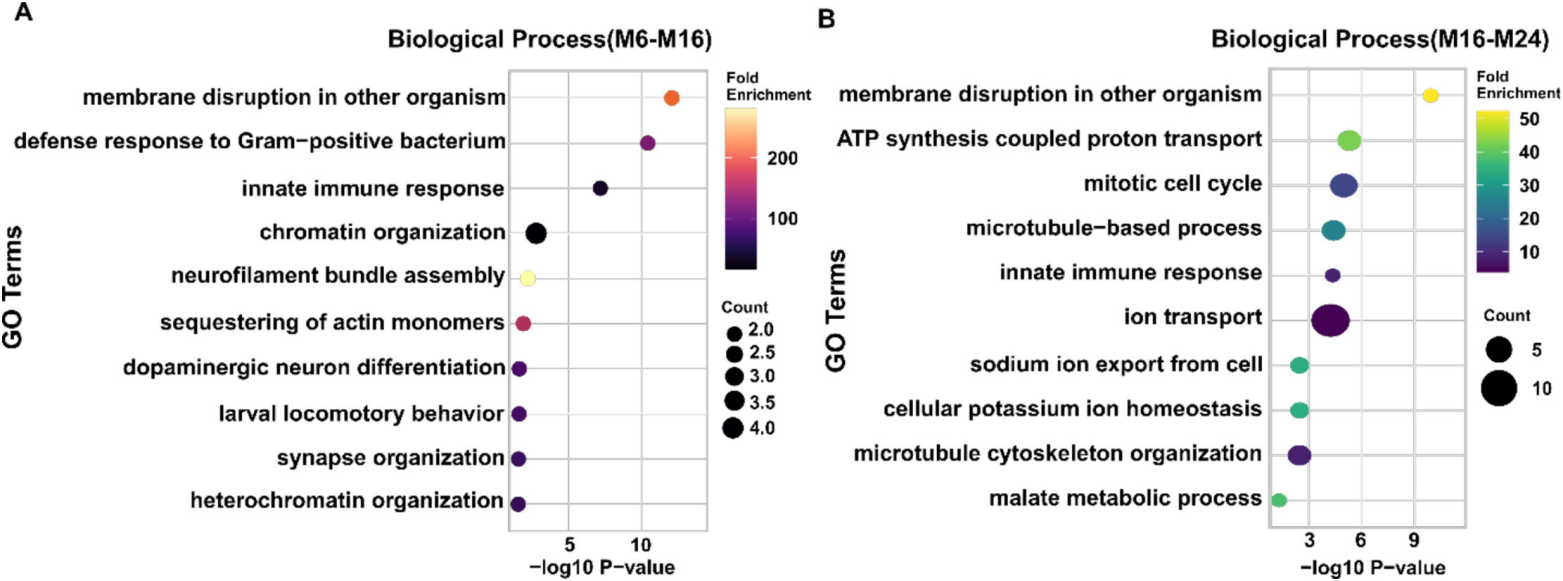
Gene Ontology (GO) enrichment analysis of differentially expressed proteoforms in males across age groups (A) M6_M16 and (B) M16_M24. The X‐axis represents the ‐log_10_(*p*‐value), indicating the significance of enrichment, while the Y‐axis lists the specific biological processes. Bubble size corresponds to the number of genes involved, and the color gradient reflects the fold enrichment, with higher values indicating stronger enrichment. The DAVID tool [71] was used for the GO analysis.

We utilized the DAVID tool [71] to perform Gene Ontology (GO) enrichment analysis, aiming to identify the most enriched biological processes associated with the differentially expressed proteoforms across age groups. In the analysis of the 6‐month‐old male brain and the 16‐month‐old male brain, the enriched biological process categories include neurofilament bundle assembly, sequestering of actin monomers, defense response to Gram‐positive bacteria, and dopaminergic neuron differentiation (Figure 3A). The process of assembling neurofilament bundles, the most abundant biological process category, involves the neurofilament light polypeptide and the neurofilament medium chain protein. The neurofilament light polypeptide contains a unique C‐terminal proteoform. A mass shift of +79.034 Da localized in the sequence of this proteoform may result from either a sequence variation or a post‐translational modification (PTM) such as phosphorylation. The neurofilament medium chain includes two truncated proteoforms. All three proteoforms show higher abundance in the 6‐month‐old male brain. The neurofilament light chain (NEFL) plays a crucial role in the structure of the neuronal cytoskeleton, forming part of neurofilaments alongside the neurofilament medium chain (NEFM) and neurofilament heavy chain (NEFH) [72, 73]. NEFL is also essential for neurofilament assembly, which regulates axonal caliber outgrowth, regeneration, guidance, and nerve impulse conduction. In ad, abnormal NEFL expression disrupts the neuronal cytoskeleton, leading to neurofibrillary tangles, structural abnormalities, cognitive decline, and memory loss [72, 73]. The process of dopaminergic neuron differentiation involves the analysis of beta‐synuclein and gamma1a‐synuclein proteins. Beta‐synuclein is characterized by a single acetylated full‐length proteoform, while gamma1a‐synuclein contains a full‐length proteoform with a mass shift of +71.94 Da in this study. Both beta‐synuclein and gamma1a‐synuclein are crucial for the proper functioning of neurons. They play key roles in maintaining synaptic activity, protecting against neurodegenerative processes, and regulating motor behavior [74, 75]. Their involvement in the development and function of dopaminergic neurons, along with their compensatory mechanisms, highlights their importance in the central nervous system (CNS) [74, 75].

In the 16‐ and 24‐month‐old male brains, the enriched biological process (BP) categories included membrane disruption in other organisms, malate metabolic process, ATP synthesis coupled proton transport, sodium ion export from the cell, and cellular potassium ion homeostasis (Figure 3B). The malate metabolic process, the second most enriched BP category, is associated with the malate dehydrogenase (MDH) protein, which includes two differentially expressed proteoforms. One MDH proteoform, spanning residues Arg328 to Ala350, was identified as a truncated form. This proteoform showed a 6.5‐fold higher abundance in 24‐month male brains compared to 16‐month male brains (*p*‐value: 10^–2.9^) and exhibited a mass shift of 37.93 Da localized between residues Glu340 and Phe347. Another truncated proteoform with the same cleavage site displayed a 2.2‐fold higher abundance in 24‐month male brains than in 16‐month male brains (*p*‐value: 10^–2.2^). Malate dehydrogenase 2 (MDH2) is a critical mitochondrial enzyme involved in the tricarboxylic acid (TCA) cycle [76]. In ad, MDH encoded by *Mdh2* is elevated by an unknown mechanism [77]. Oxidative stress, an early and pervasive event in ad, has been shown to increase MDH activity by 19% and *mdh2* mRNA levels by 22% in the mouse hippocampal cell line HT22 [77]. Next, sodium ion export from the cell involved 14 distinct proteoforms of the sodium/potassium‐transporting ATPase subunit alpha (α3Na+/K+‐ATPase), which showed higher abundance in the brains of 24‐month‐old male zebrafish. The α3Na+/K+‐ATPase isoforms are predominantly expressed in neurons, where they play a critical role in maintaining the resting membrane potential and regulating neuronal excitability. Research in zebrafish has emphasized the vital role of α3Na+/K+‐ATPase isoforms in proper brain development [78]. Dysfunction of α3Na+/K+‐ATPase has been associated with various neurological disorders, including epilepsy, stress‐induced motor symptoms, anxiety, depression‐like behaviors, and movement disorders [78].

In male zebrafish at 6‐ and 24‐month age groups, significant enrichment was observed in two biological process categories: microtubule‐based processes and glycolytic processes (Figure S4). The microtubule‐based process category showed an increased abundance of four truncated α‐tubulin proteoforms and three β‐tubulin proteoforms, two of which are N‐terminally truncated. All seven proteoforms exhibited higher abundance in the 24‐month male sample. Microtubules are critical for neuronal function, playing key roles in intracellular transport, synaptic plasticity, and neuronal repair [79]. These dynamic structures, composed of α‐ and β‐tubulin heterodimers, undergo continuous polymerization cycles to meet cellular demands. Mutations in tubulin, particularly in αβ‐dimers and γ‐tubulin, have been linked to brain malformations and cognitive dysfunction [80, 81]. The glycolytic process category included several proteins with distinct proteoforms: glyceraldehyde‐3‐phosphate dehydrogenase 2 (GAPDH2) with three truncated proteoforms, phosphopyruvate hydratase with one truncated proteoform, pyruvate kinase with two proteoforms (one acetylated), triosephosphate isomerase B (TPIB) with one proteoform, and fructose‐bisphosphate aldolase C‐B (ALDOC‐B) with three proteoforms (two acetylated). Among these, two truncated GAPDH2 proteoforms showed differential expression between the 6‐month and 24‐month male samples, while most other proteoforms exhibited higher abundance in the 24‐month male samples. Glycolysis plays a vital role in brain energy production, synaptic transmission, and redox homeostasis [82]. Impaired glycolytic flux has been strongly associated with ad pathology, particularly in relation to amyloid‐beta and tau accumulation [83].

Gene Ontology (GO) analysis of female samples revealed significant enrichment in heterochromatin and chromatin organization processes at 6 and 16 months (Figure S5). The heterochromatin organization category included six H2A proteoforms. Among these, two were full‐length proteoforms with distinct expression patterns—one highly expressed in 6‐month‐old females and another acetylated proteoform with higher abundance in 16‐month‐old females. The remaining proteoforms were C‐terminally truncated and exhibited higher abundance in 16‐month‐old females. Chromatin organization processes involved HMGN2 (five full‐length proteoforms, including one acetylated), HMGN6 (two full‐length proteoforms), and non‐histone chromosomal protein HMG‐17. HMGN2 plays a role in numerous cellular processes, including chromatin structure regulation, transcription, DNA repair, antimicrobial activity, cell homing, and cytokine release regulation [84, 85, 86, 87]. Studies have shown that acetylated HMGN2 has reduced nucleosome binding affinity compared to non‐acetylated forms, suggesting that acetylation may weaken HMGN2‐nucleosome interactions [88]. High mobility group box (HMGB) proteins are critical regulators of aging, influencing chromatin dynamics, gene expression, and the senescence‐associated secretory phenotype (SASP) [89, 90]. As damage‐associated molecular patterns (DAMPs), HMGB proteins promote inflammation, contributing to age‐related diseases such as cardiovascular disorders, cancer, and type 2 diabetes [91, 92].

Analysis of female specimens at 6 and 24 months revealed significant enrichment in hydrolase activity acting on carbon‐nitrogen bonds (excluding peptide bonds). This process involved Collapsin response mediator protein 5.1 (CRMP5) and Dihydropyrimidinase‐related protein 2 (DRP‐2) (Figure S6). CRMP5 displayed two proteoforms: one N‐terminally acetylated and one truncated, both exhibiting increased abundance in 24‐month female samples. CRMPs are intracellular phosphoproteins critical for neuronal growth cone progression and migration [93, 94, 95, 96, 97]. They localize to neuronal lamellipodia, filopodia, axon shafts, and cell bodies, where they facilitate axon formation by binding tubulin heterodimers and promoting microtubule assembly [98, 99]. Additionally, CRMP5 autoantibodies have been identified as key biomarkers for various conditions, including paraneoplastic neurologic autoimmunity [100], myelopathy [101], optic neuritis, vitreitis, retinitis [102, 103, 104], and cancer diagnosis [105]. DRP‐2 exhibited two truncated proteoforms with elevated expression in 24‐month female samples. DRP‐2 regulates dendritic length and supports microtubule assembly and cytoskeletal remodeling. Shortened dendritic lengths observed in ad brains may impair neuronal communication [106, 107]. Furthermore, DRP‐2's interaction with neurofibrillary tangles reduces its cytosolic levels [108, 109], and its decreased expression in Down syndrome patients suggests a role for amyloid‐beta (Aβ) in its regulation [108]. The correlation between ad‐related memory loss and reduced DRP‐2 levels underscores its significance [107].

Synaptic vesicle priming emerged as a significantly enriched biological process in female 16‐ and 24‐month comparisons (Figure S7). This process is facilitated by two key proteins: Synaptosomal‐Associated Protein 25‐A (SNAP‐25A) and SNAP‐25B. SNAP‐25A exhibited two N‐terminal truncated proteoforms with 5.3‐ and 2.4‐fold higher abundance in 24‐month‐old female brains compared to 16‐month‐old female brains. Additionally, SNAP‐25B showed one N‐terminal truncated proteoform with elevated abundance in the 24‐month‐old female brain. SNAP‐25 is a critical component of the trimeric SNARE complex, playing an essential role in vesicular exocytosis. It supports neuronal growth, dense core vesicle secretion, and synaptic vesicle release [110]. Dysregulation of SNAP‐25 has been linked to multiple neurodegenerative conditions, including Huntington's disease, ad, and diabetes [111, 112]. The protein's variable expression across synapses influences calcium sensitivity and synaptic activity, underscoring its importance in neurotransmission modulation [111, 112]. Furthermore, multiple studies have reported elevated cerebrospinal fluid (CSF) levels of SNAP‐25 in ad patients [113, 114, 115, 116, 117]. This highlights the potential of SNAP‐25 proteoforms as biomarkers of ad.

### 3.2 Quantitative TDP Analysis of Zebrafish Male and Female Brains as a Function of Age Discovered Potentially Critical Proteoforms Modulating Sexual Dimorphism of Brain Aging

The sexual dimorphism of brains is primarily influenced by the expression of sex chromosome genes and the impact of hormones produced by the gonads. This dimorphism plays a crucial role in determining the phenotypic variations in memory, cognition, emotion, stress response, and reproductive behaviors [118]. Here, we performed the first quantitative TDP study of brains regarding sexual dimorphism during the aging process in the brain using the zebrafish model.

**FIGURE 4:**
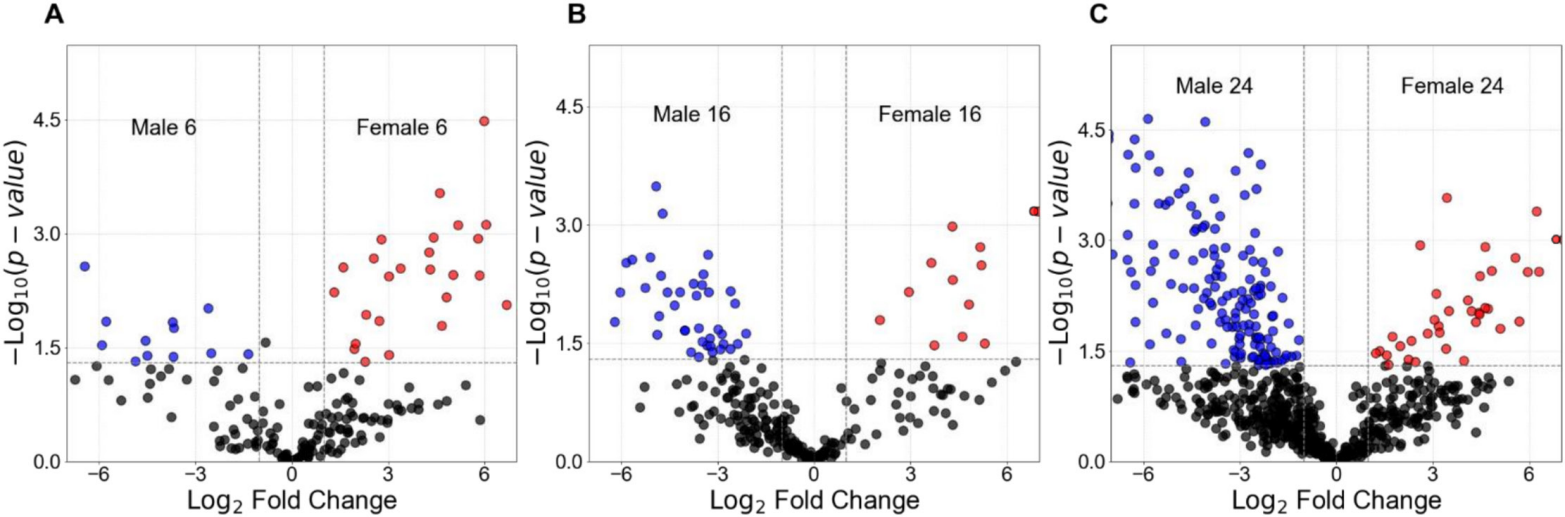
Quantitative TDP analysis of female and male zebrafish brains of the same age but different genders, using CZE‐MS/MS. The volcano plots show the ‐log(*p*‐value) against the log2(fold change, female/male) for quantified proteoforms at different ages: (A) 6 months old, (B) 16 months old, and (C) 24 months old. The vertical and horizontal dotted lines show the log2 fold‐change cut‐off of ± 1 and of *p* = 0.05, respectively. Proteoforms with higher abundance in female brains are highlighted in red, while those more abundant in male brains are highlighted in blue. The volcano plot was generated using Python software (Version 3.11.4) [65].

In this study, 185, 261, and 750 proteoforms were quantified between male and female zebrafish brains at three different ages, 6‐, 16‐, and 24 months, respectively. Out of those proteoforms, 38, 54, and 207 proteoforms of 27, 40, and 123 proteins showed statistically significant differences in abundance between male and female brains at 6, 16, and 24 months (Figure 4A–C). The differentially expressed proteoforms are listed in Supporting Information II.

To comprehend the biological importance of these distinctively expressed proteoforms, we conducted GO enrichment analysis on genes whose proteoforms exhibited significantly higher abundance in the brains of females and males in the same age groups.

GO analysis comparing male and female specimens at 6 months (Figure S8) identified enrichment in oxygen transport processes, containing Alpha globin‐like and Hemoglobin subunit beta‐2 proteins. The Alpha globin‐like protein displayed an N‐terminally acetylated proteoform, while the Hemoglobin subunit beta‐2 exhibited an N‐terminal proteoform. Both proteoforms showed higher abundance in the brain of 6‐month‐old females. Although hemoglobin is primarily known for its role as an oxygen carrier in vertebrate erythrocytes, recent studies have highlighted its expression in neurons and its sensitivity to mitochondrial‐targeting treatments [119].

GO analysis comparing male and female specimens at 16 months of age (Figure S9) revealed axonogenesis as an enriched biological process. This process involved two key proteins: Microtubule‐associated protein 1A isoform X2, which exhibited one truncated proteoform, and Non‐histone chromosomal protein HMG‐17, also known as High Mobility Group Nucleosome‐binding protein 17 (HMGN‐17), which displayed two C‐terminally truncated proteoforms. Both proteins showed higher abundance in the brain of 16‐month‐old females than in the brain of males of the same age. Axonogenesis is a critical process that drives the creation and extension of axons, establishing neuronal polarity and functional morphology [120]. HMGN proteins play an essential regulatory role in this process through their chromatin‐modifying activities [121]. These proteins compete with histone H1 for AT‐rich chromatin regions, altering DNA compaction and influencing the transcription of genes required for axonal development [121, 122, 123, 124]. This regulatory mechanism appears to have sex‐specific effects, as shown by notable differences in hypothalamic neuronal development between males and females [125].

In 24‐month‐old female and male brains (Figure S10), we identified enriched biological process categories, including phosphocreatine biosynthetic process, sequestering of actin monomers, synaptic vesicle priming, ATP metabolic process, and sodium ion export from cells. The phosphocreatine biosynthetic process stood out because of the involvement of five distinct truncated proteoforms of creatine kinase (CK), which displayed sexually dimorphic expression patterns. Four proteoforms were upregulated in the brain of 24‐month‐old males, while one proteoform showed a substantial upregulation in the brain of 24‐month‐old females, with a fivefold change (*p*‐value: 10^−2^·^5^). CK plays a vital role in maintaining cellular energy balance by catalyzing the transfer of a phosphate group from ATP to creatine, forming phosphocreatine and ADP [126, 127]. This process is essential for stabilizing and transporting energy in cells with high energy demands, such as muscle cells and neurons [126, 127]. Our findings support the established hypothesis of sexual dimorphism in CK expression, where the brains of males typically exhibit higher CK levels compared to females. This difference is primarily attributed to sex‐based variations in muscle mass and the influence of sex hormones, particularly testosterone, on muscle metabolism. These factors contribute to greater CK release into the bloodstream following muscle tissue damage in males compared to females [128, 129, 130]. Thymosin beta (Tβ) was implicated in the second most enriched biological process category, the sequestering of actin monomers. Two full‐length proteoforms of Tβ showed significantly higher abundance in female brains. Research on thymosin beta in zebrafish has demonstrated its ability to bind to monomeric actin, highlighting its role in regulating neuronal growth and differentiation [30, 131].

Overall, these findings reveal significant sex‐related variations in proteoform expression across males and females, highlighting the complexity and dynamic nature of sexual dimorphism in brain proteomes. Understanding these differences at the proteoform level provides deeper insights into how sex influences brain function and development, contributing to phenotypic differences in memory, cognition, emotion, stress response, and reproductive behaviors.

## 4 Conclusions

This study presents a detailed analysis of age‐related changes (6, 16, and 24 months) in the brain proteoforms of male and female zebrafish using CZE‐MS/MS in TDP. This comprehensive proteoform‐level analysis revealed significant age‐dependent and sex‐specific changes in brain proteoforms. This study identified crucial proteoform modifications in proteins associated with neuronal structure, metabolism, and synaptic function. Key findings include age‐related changes in neurofilament proteins, synaptic proteins like SNAP‐25, and metabolic enzymes such as malate dehydrogenase. Sexual dimorphism was evident in the expression of proteins involved in oxygen transport, axonogenesis, and energy metabolism, with notable differences in creatine kinase proteoforms between males and females at 24 months. Additionally, we identified a truncated proteoform of AP2B1, a subunit of the AP2 adaptor complex, in female zebrafish aged 24 months, suggesting potential age‐ and sex‐specific roles in brain aging and neurodegeneration. These findings provide valuable insights into the molecular mechanisms underlying brain aging and sexual dimorphism, establishing a foundation for future investigations into age‐related neurological conditions and sex‐specific therapeutic approaches.

## Funding

This work was supported by the National Institute of General Medical Sciences (R35GM153479) and the National Cancer Institute (R01CA247863).

## Conflicts of Interest

The authors declare no conflicts of interest.

## Supporting information


**Figure S1:** Pearson's correlation coefficients of label‐free quantification intensities of overlapped proteoforms between any two technical replicates of the 16‐month‐old male zebrafish brain. The color code is based on their Pearson's correlation coefficient values from −1 (red) to 1 (blue).
**Figure S2:** Amino acid sequences and MS/MS fragmentation patterns of one identified proteoforms. AP2B1 proteoform featuring truncated proteoform, and mass shifts of 250.125 Da. The marked amino acid residue regions indicate the potential modification sites. The exact modification sites cannot be determined in most cases because of the limited backbone cleavage coverage.
**Figure S3:** Phosphorylated proteoforms show age‐dependent expression changes in female zebrafish brains. Violin plots with overlaid boxplots illustrate the abundance of four phosphorylated proteoforms—Hmgn6, Calm1a, Dpysl3, and Nfma—across female brain samples at 6, 16, and 24 months (F6, F16, and F24). Each proteoform shows an about +80‐Da mass shift consistent with phosphorylation. Hmgn6(2–93), Calm1a (2–149), Dpysl3 (512–551), and Nfma (565–615) display distinct age‐associated abundance patterns, suggesting dynamic phosphorylation events during brain aging. *p < 0.05; **p < 0.01; ***p < 0.001.
**Figure S4:**. Gene Ontology (GO) enrichment analysis using DAVID tool [1] of differentially expressed proteoforms in the brain of 6‐ and 24‐month‐old males. The X‐axis represents the ‐log_10_(*p*‐value), indicating the significance of enrichment, while the Y‐axis lists the specific biological processes. Bubble size corresponds to the number of genes involved, and the color gradient reflects the fold enrichment, with higher values indicating stronger enrichment.
**Figure S5:**. Gene Ontology (GO) enrichment analysis using DAVID tool [1] of differentially expressed proteoforms in the brain of 6‐ and 16‐month‐old females. The X‐axis represents the ‐log_10_(*p*‐value), indicating the significance of enrichment, while the Y‐axis lists the specific biological processes. Bubble size corresponds to the number of genes involved, and the color gradient reflects the fold enrichment, with higher values indicating stronger enrichment.
**Figure S6:**. Gene Ontology (GO) enrichment analysis using DAVID tool [1] of differentially expressed proteoforms in the brain of 6‐ and 24‐month‐old females. The X‐axis represents the ‐log_10_(*p*‐value), indicating the significance of enrichment, while the Y‐axis lists the specific biological processes. Bubble size corresponds to the number of genes involved, and the color gradient reflects the fold enrichment, with higher values indicating stronger enrichment.
**Figure S7:**. Gene Ontology (GO) enrichment analysis using DAVID tool [1] of differentially expressed proteoforms in the brain of 16‐ and 24‐month‐old females. The X‐axis represents the ‐log_10_(*p*‐value), indicating the significance of enrichment, while the Y‐axis lists the specific biological processes. Bubble size corresponds to the number of genes involved, and the color gradient reflects the fold enrichment, with higher values indicating stronger enrichment.
**Figure S8:**. Gene Ontology (GO) enrichment analysis using DAVID tool [1] of differentially expressed proteoforms in the 6‐month‐old female and male brains. The X‐axis represents the ‐log_10_(*p*‐value), indicating the significance of enrichment, while the Y‐axis lists the specific biological processes. Bubble size corresponds to the number of genes involved, and the color gradient reflects the fold enrichment, with higher values indicating stronger enrichment.
**Figure S9:**. Gene Ontology (GO) enrichment analysis using DAVID tool [1] of differentially expressed proteoforms in the 16‐month‐old female and male brains. The X‐axis represents the ‐log_10_(*p*‐value), indicating the significance of enrichment, while the Y‐axis lists the specific biological processes. Bubble size corresponds to the number of genes involved, and the color gradient reflects the fold enrichment, with higher values indicating stronger enrichment.
**Figure S10:**. Gene Ontology (GO) enrichment analysis using DAVID tool [1] of differentially expressed proteoforms in the 24‐month‐old female and male brains. The X‐axis represents the ‐log_10_(*p*‐value), indicating the significance of enrichment, while the Y‐axis lists the specific Biological Processes. Bubble size corresponds to the number of genes involved, and the color gradient reflects the fold enrichment, with higher values indicating stronger enrichment.

**Data S1:** Supporting Information.

## Data Availability

The mass spectrometry proteomics data have been deposited to the ProteomeXchange Consortium via the PRIDE [132] partner repository with the dataset identifier PXD060426.
