# Supplementary material for: Top‐Down Proteomics Study of Aging and Sexual Dimorphism of Zebrafish Brains
Source: J Mass Spectrom. 2025 Dec 30;61(1):e70009. doi: 10.1002/jms.70009 (PMC12750134; doi:10.1002/jms.70009)
Supplement: Supplementary file 1 — Figure S1: Pearson's correlation coefficients of label‐free quantification intensities of overlapped proteoforms between any two technical replicates of the 16‐month‐old male zebrafish brain. The color code is based on their Pearson's correlation coefficient values from −1 (red) to 1 (blue). Figure S2: Amino acid sequences and MS/MS fragmentation patterns of one identified proteoforms. AP2B1 proteoform featuring truncated proteoform, and mass shifts of 250.125 Da. The marked amino acid residue regions indicate the potential modification sites. The exact modification sites cannot be determined in most cases because of the limited backbone cleavage coverage. Figure S3: Phosphorylated proteoforms show age‐dependent expression changes in female zebrafish brains. Violin plots with overlaid boxplots illustrate the abundance of four phosphorylated proteoforms—Hmgn6, Calm1a, Dpysl3, and Nfma—across female brain samples at 6, 16, and 24 months (F6, F16, and F24). Each proteoform shows an about +80‐Da mass shift consistent with phosphorylation. Hmgn6(2–93), Calm1a (2–149), Dpysl3 (512–551), and Nfma (565–615) display distinct age‐associated abundance patterns, suggesting dynamic phosphorylation events during brain aging. *p < 0.05; **p < 0.01; ***p < 0.001. Figure S4:. Gene Ontology (GO) enrichment analysis using DAVID tool [1] of differentially expressed proteoforms in the brain of 6‐ and 24‐month‐old males. The X‐axis represents the ‐log10(p‐value), indicating the significance of enrichment, while the Y‐axis lists the specific biological processes. Bubble size corresponds to the number of genes involved, and the color gradient reflects the fold enrichment, with higher values indicating stronger enrichment. Figure S5:. Gene Ontology (GO) enrichment analysis using DAVID tool [1] of differentially expressed proteoforms in the brain of 6‐ and 16‐month‐old females. The X‐axis represents the ‐log10(p‐value), indicating the significance of enrichment, while the Y‐axis lists the sp [file JMS-61-e70009-s002.docx]

**Supporting Information I**

**Top-down proteomics study of aging and sexual dimorphism of**

**zebrafish brains**

Mehrdad Falamarzi Askarani,^1^ William Poulos,^2^ Maryam Rahimzadeh Dashtaki,^1^ Seyed Amirhossein Sadeghi,^1^ Jose B. Cibelli,^2,3^ Fei Fang,^1,^* Liangliang Sun^1,^*

^1^Department of Chemistry, Michigan State University, 578 S Shaw Lane, East Lansing, Michigan 48824, USA

^2^Department of Animal Science, Michigan State University, East Lansing, MI 48824, USA.

^3^Department of Large Animal Clinical Sciences, Michigan State University, East Lansing, MI 48824, USA.

* Corresponding Authors.

F. Fang, Email: fangfei1@msu.edu

L. Sun, Email: lsun@chemistry.msu.edu; Phone: 517-353-0498


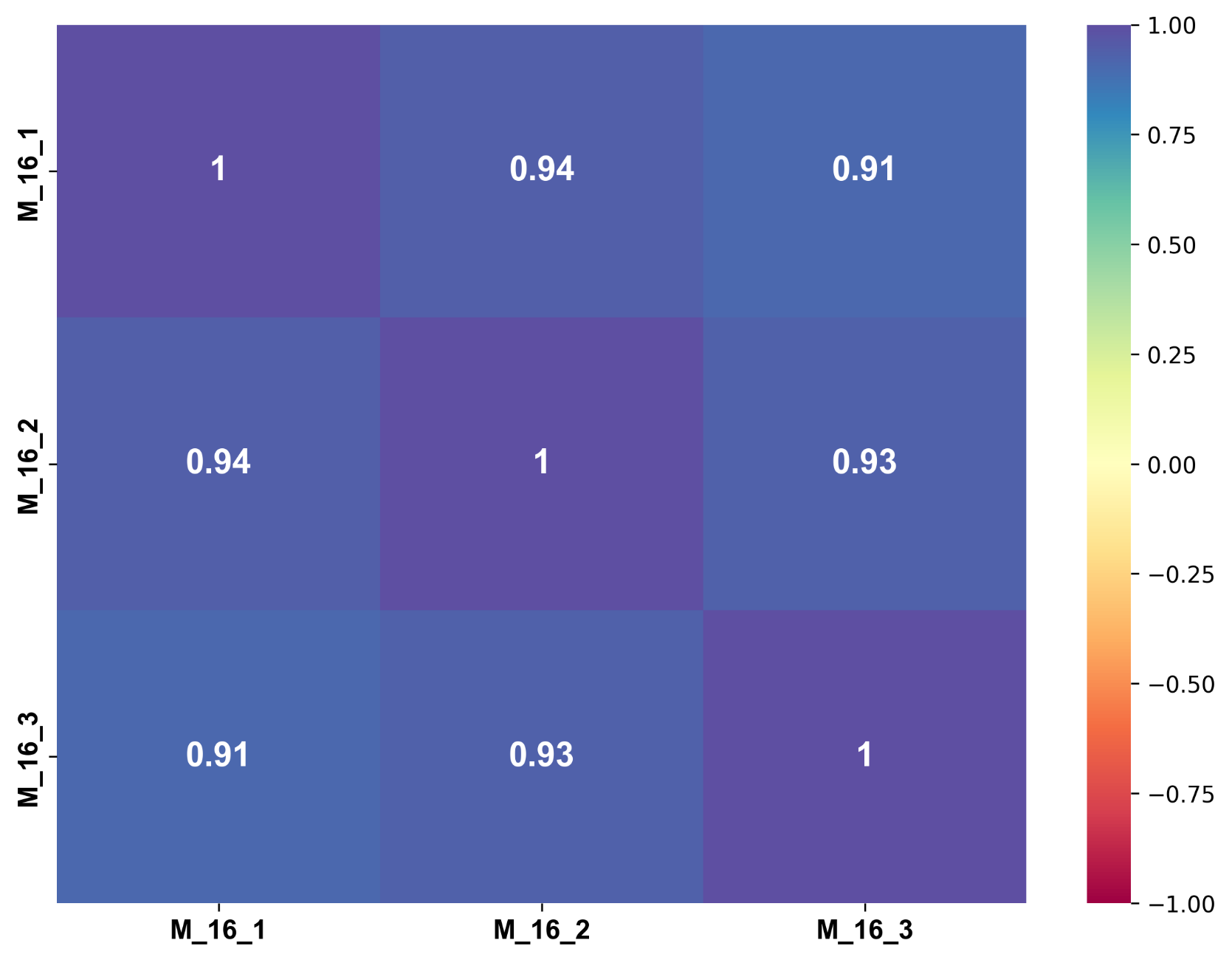


**Figure S1.** Pearson’s correlation coefficients of label-free quantification intensities of overlapped proteoforms between any two technical replicates of the 16-month-old male zebrafish brain. The color code is based on their Pearson’s correlation coefficient values from −1 (red) to 1 (blue).


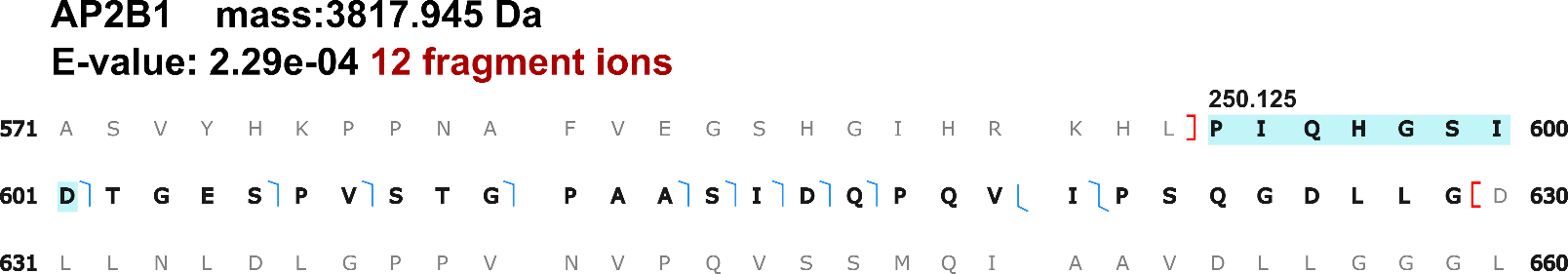


**Figure S2.** Amino acid sequences and MS/MS fragmentation patterns of one identified proteoforms. AP2B1 proteoform featuring truncated proteoform, and mass shifts of 250.125 Da. The marked amino acid residue regions indicate the potential modification sites. The exact modification sites cannot be determined in most cases due to the limited backbone cleavage coverage.


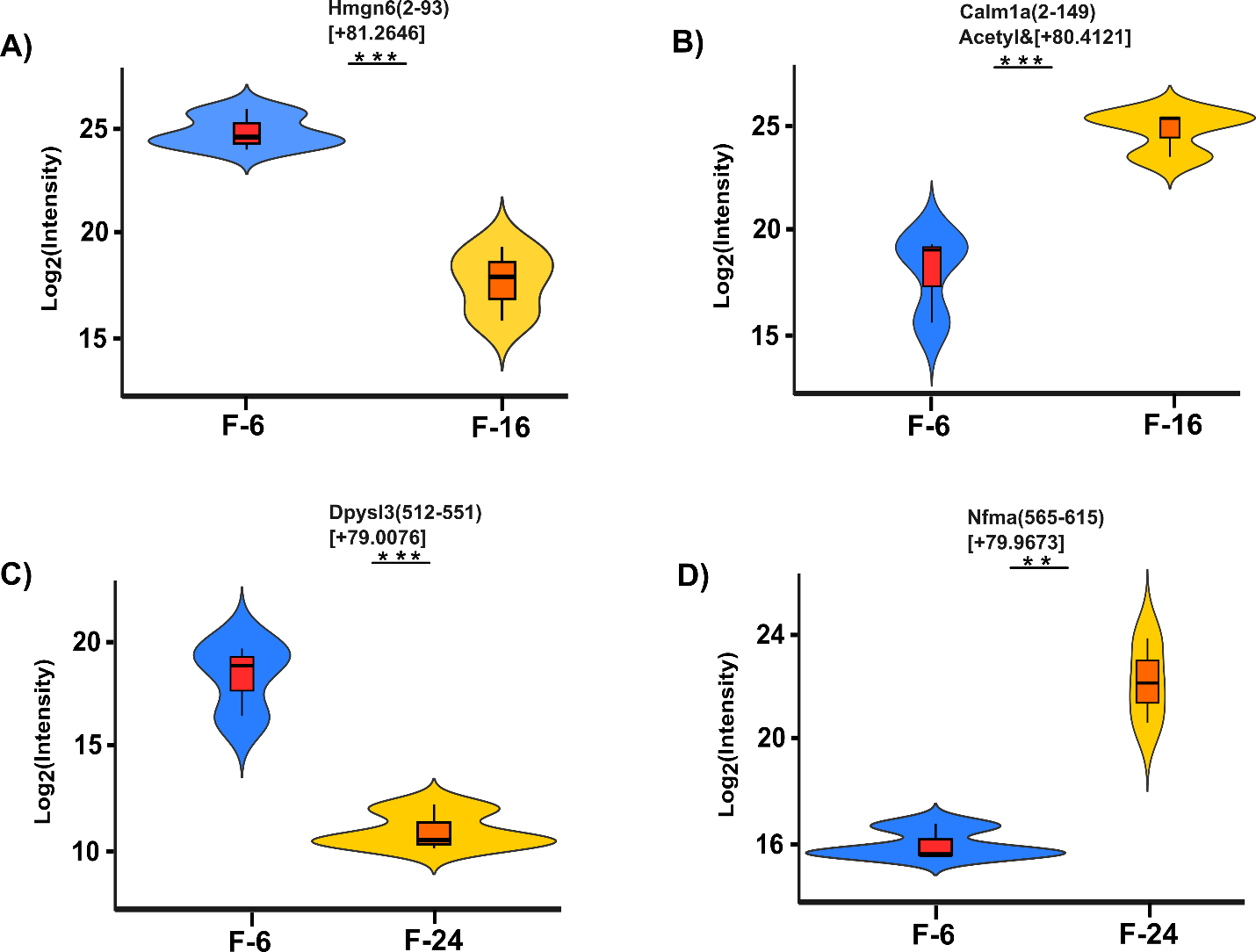


**Figure S3.** Phosphorylated proteoforms show age-dependent expression changes in female zebrafish brains. Violin plots with overlaid boxplots illustrate the abundance of four phosphorylated proteoforms—Hmgn6, Calm1a, Dpysl3, and Nfma—across female brain samples at 6, 16, and 24 months (F6, F16, F24). Each proteoform shows an about +80- Da mass shift consistent with phosphorylation. Hmgn6(2–93), Calm1a (2–149), Dpysl3 (512–551), and Nfma (565–615) display distinct age-associated abundance patterns, suggesting dynamic phosphorylation events during brain aging. **p* < 0.05; ** *p* < 0.01; *** *p* < 0.001.

**
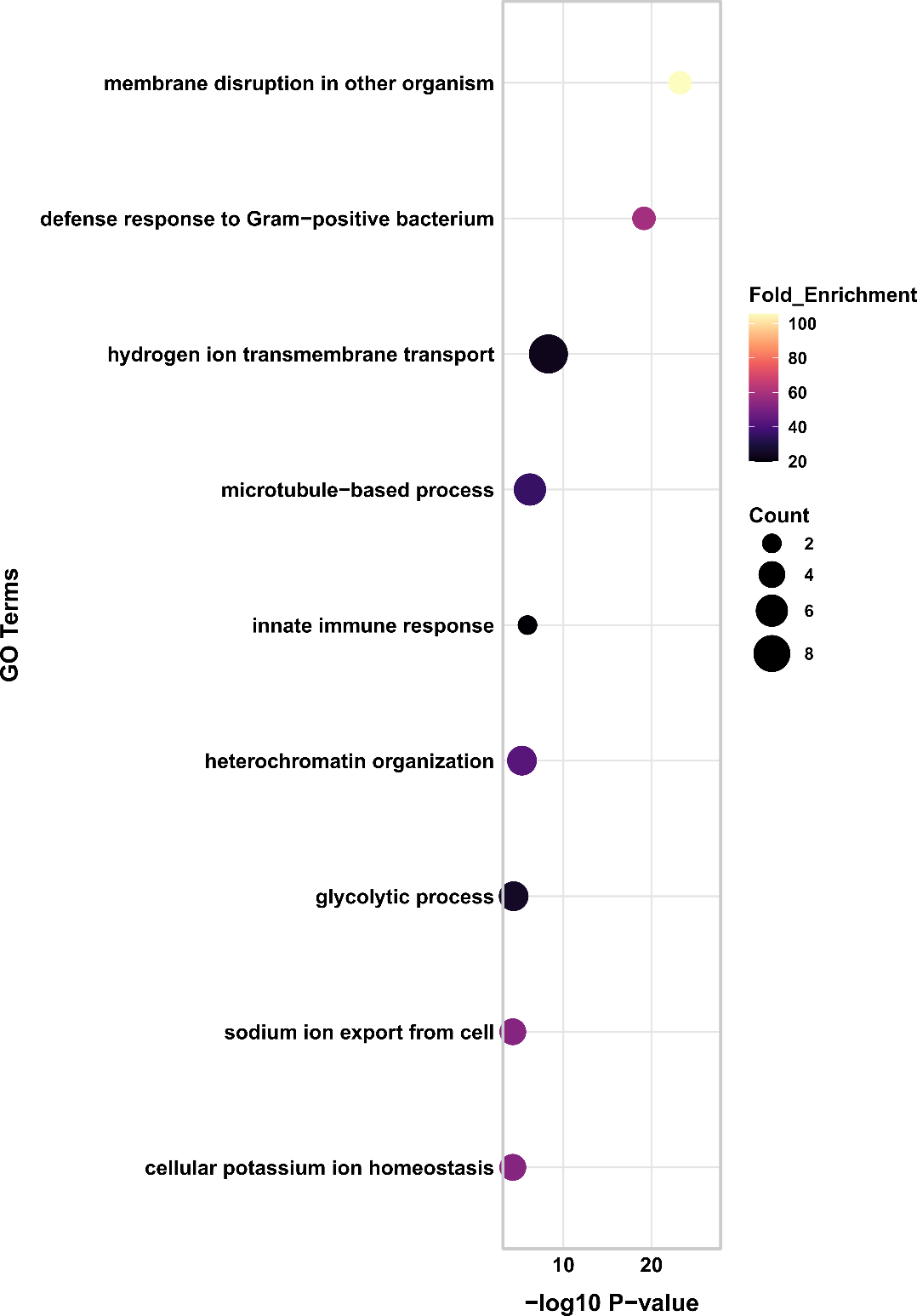
Figure S4**. Gene Ontology (GO) enrichment analysis using DAVID tool [1] of differentially expressed proteoforms in the brain of 6- and 24-month-old males. The X-axis represents the -log₁₀(p-value), indicating the significance of enrichment, while the Y-axis lists the specific Biological Processes. Bubble size corresponds to the number of genes involved, and the color gradient reflects the fold enrichment, with higher values indicating stronger enrichment.


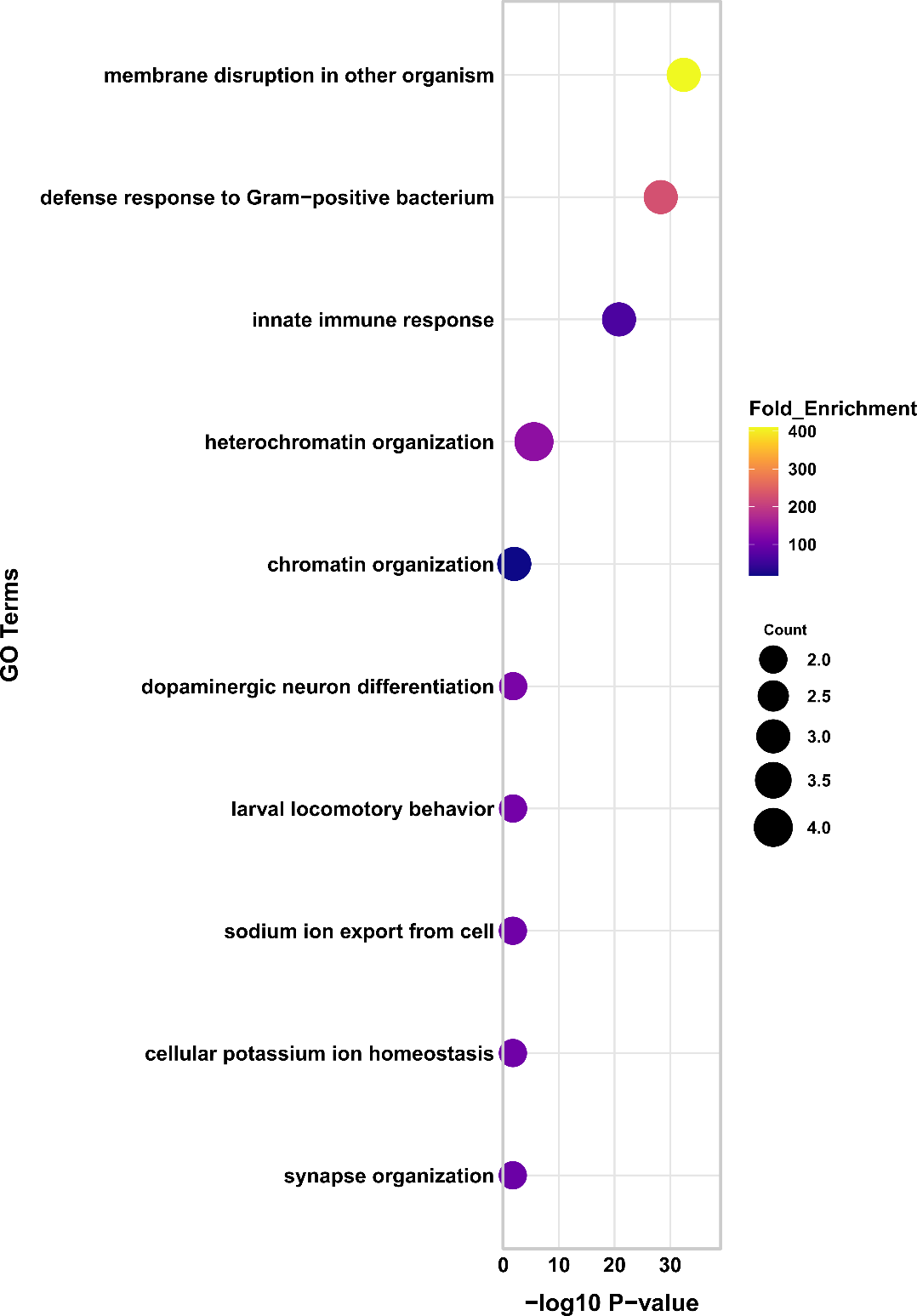


**Figure S5**. Gene Ontology (GO) enrichment analysis using DAVID tool [1] of differentially expressed proteoforms in the brain of 6- and 16-month-old females. The X-axis represents the -log₁₀(p-value), indicating the significance of enrichment, while the Y-axis lists the specific Biological Processes. Bubble size corresponds to the number of genes involved, and the color gradient reflects the fold enrichment, with higher values indicating stronger enrichment.


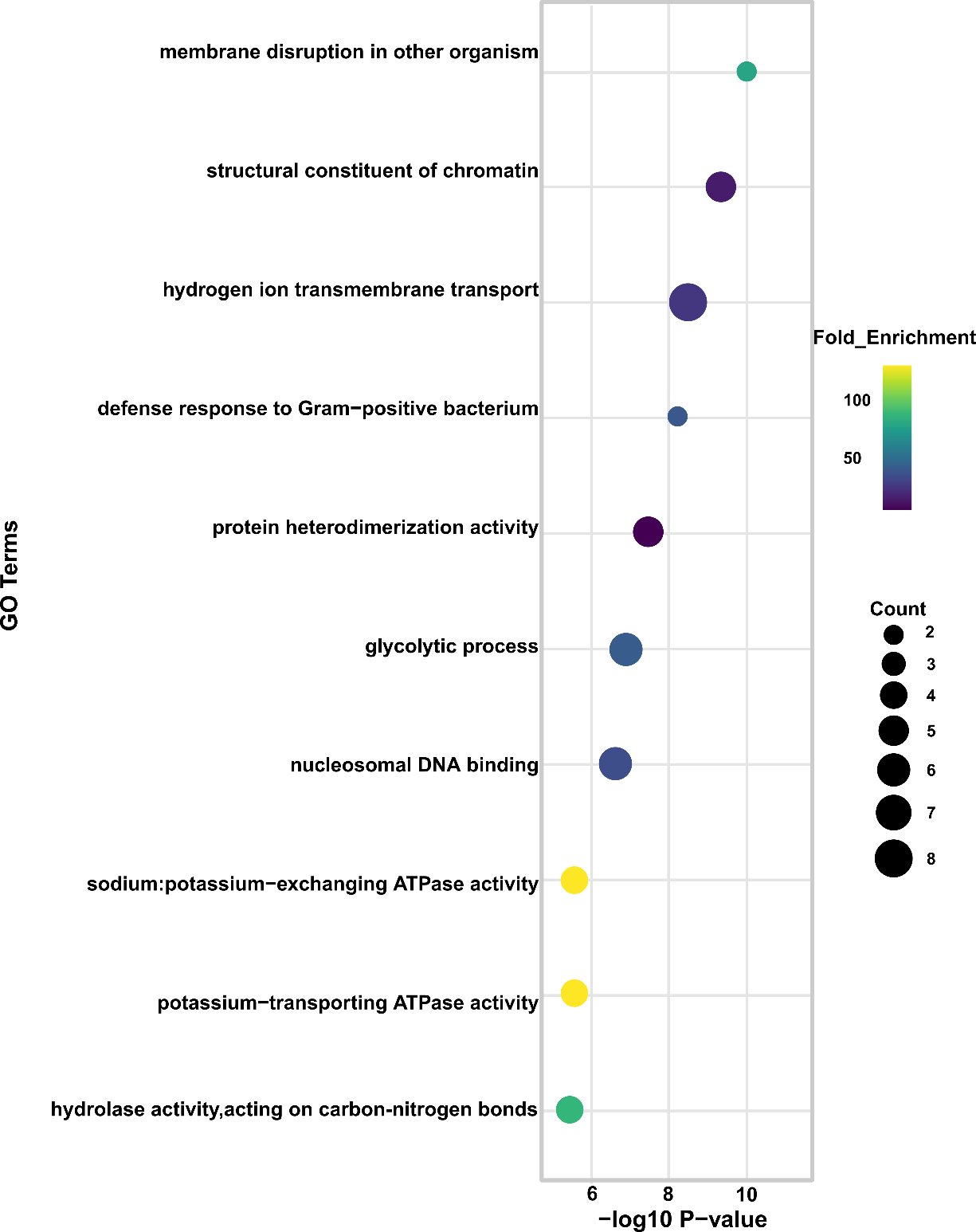


**Figure S6**. Gene Ontology (GO) enrichment analysis using DAVID tool [1] of differentially expressed proteoforms in the brain of 6- and 24-month-old females. The X-axis represents the -log₁₀(p-value), indicating the significance of enrichment, while the Y-axis lists the specific Biological Processes. Bubble size corresponds to the number of genes involved, and the color gradient reflects the fold enrichment, with higher values indicating stronger enrichment.


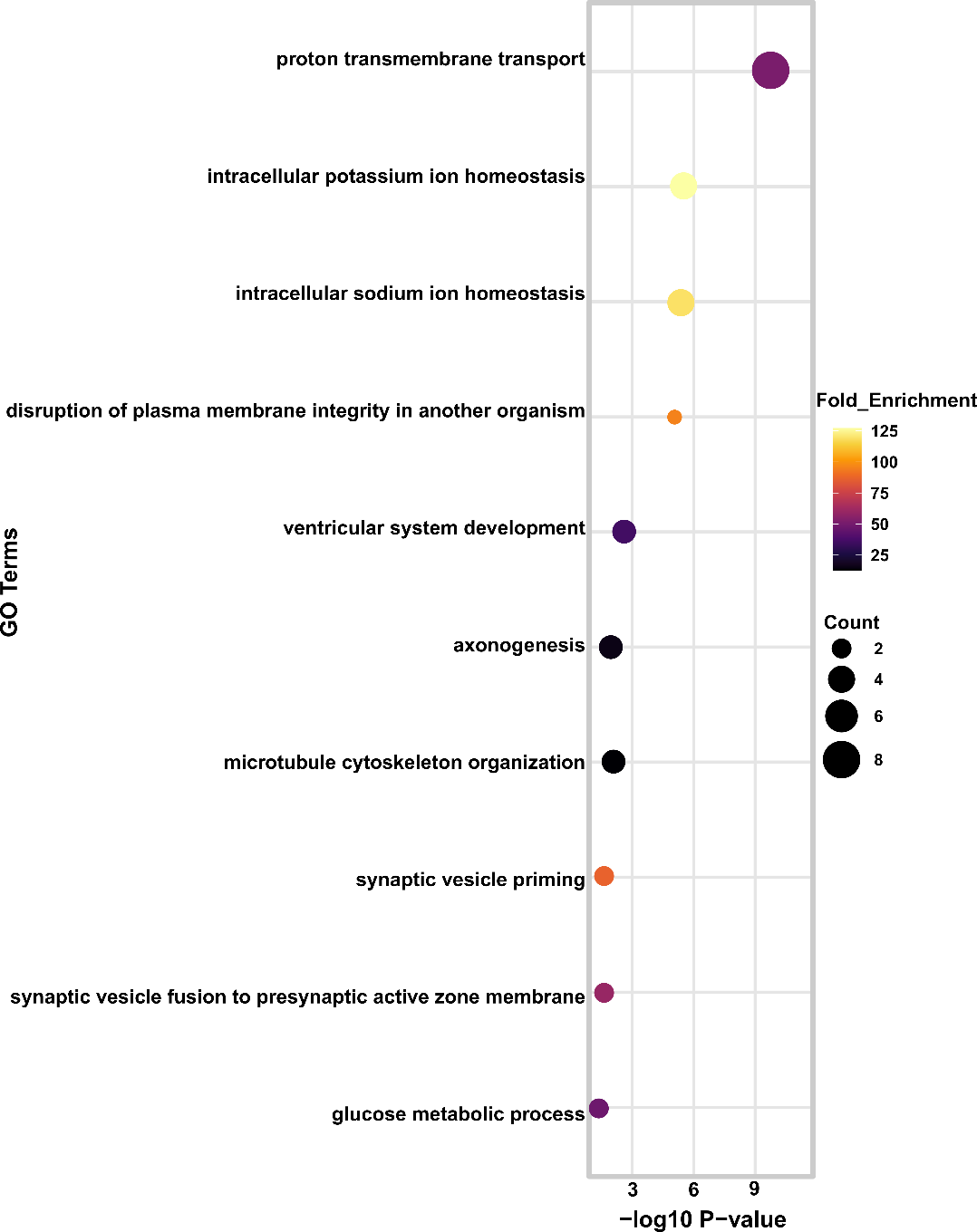


**Figure S7**. Gene Ontology (GO) enrichment analysis using DAVID tool [1] of differentially expressed proteoforms in the brain of 16- and 24-month-old females. The X-axis represents the -log₁₀(p-value), indicating the significance of enrichment, while the Y-axis lists the specific Biological Processes. Bubble size corresponds to the number of genes involved, and the color gradient reflects the fold enrichment, with higher values indicating stronger enrichment.


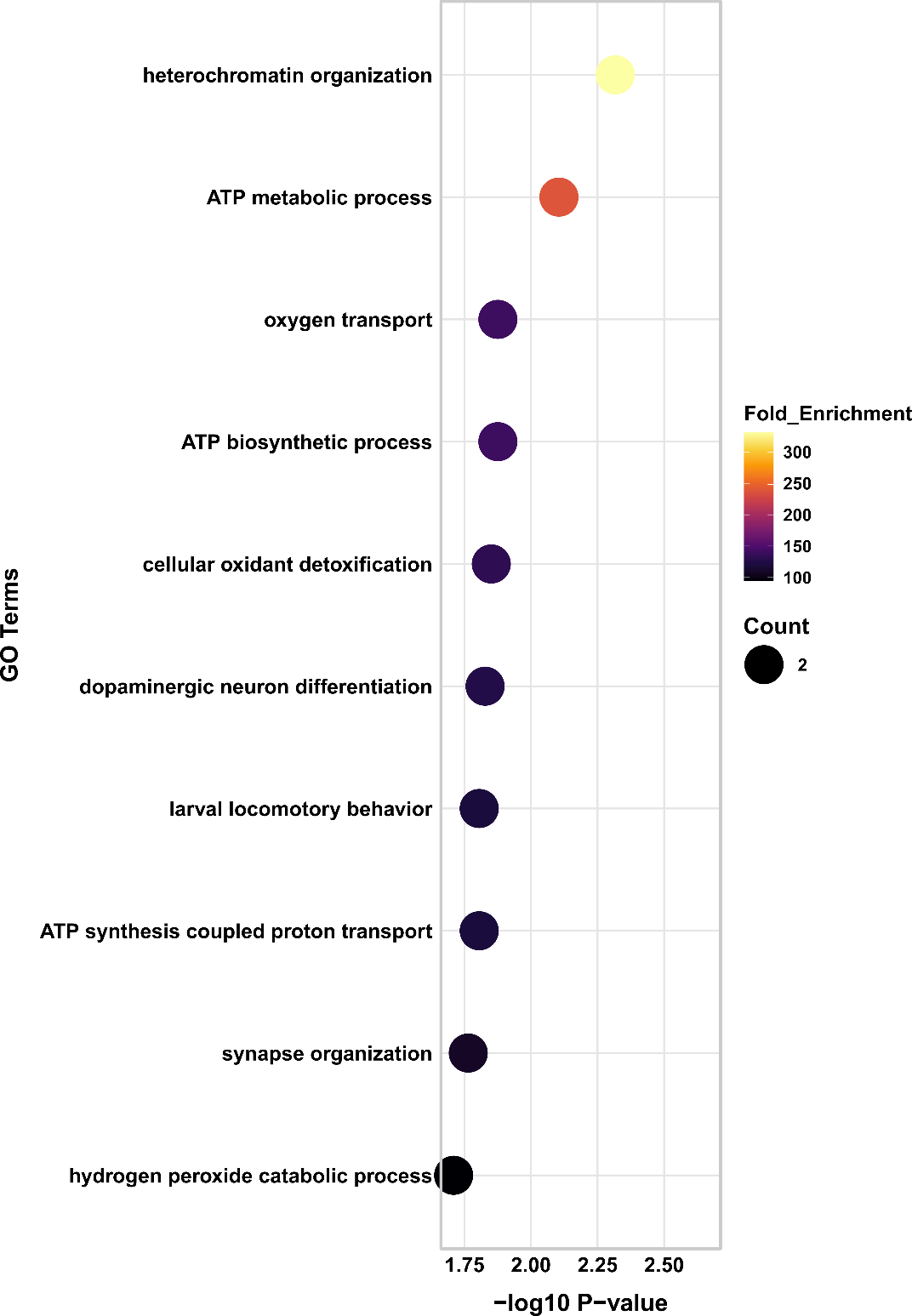


**Figure S8**. Gene Ontology (GO) enrichment analysis using DAVID tool [1] of differentially expressed proteoforms in the 6-month-old female and male brains. The X-axis represents the -log₁₀(p-value), indicating the significance of enrichment, while the Y-axis lists the specific Biological Processes. Bubble size corresponds to the number of genes involved, and the color gradient reflects the fold enrichment, with higher values indicating stronger enrichment.

**
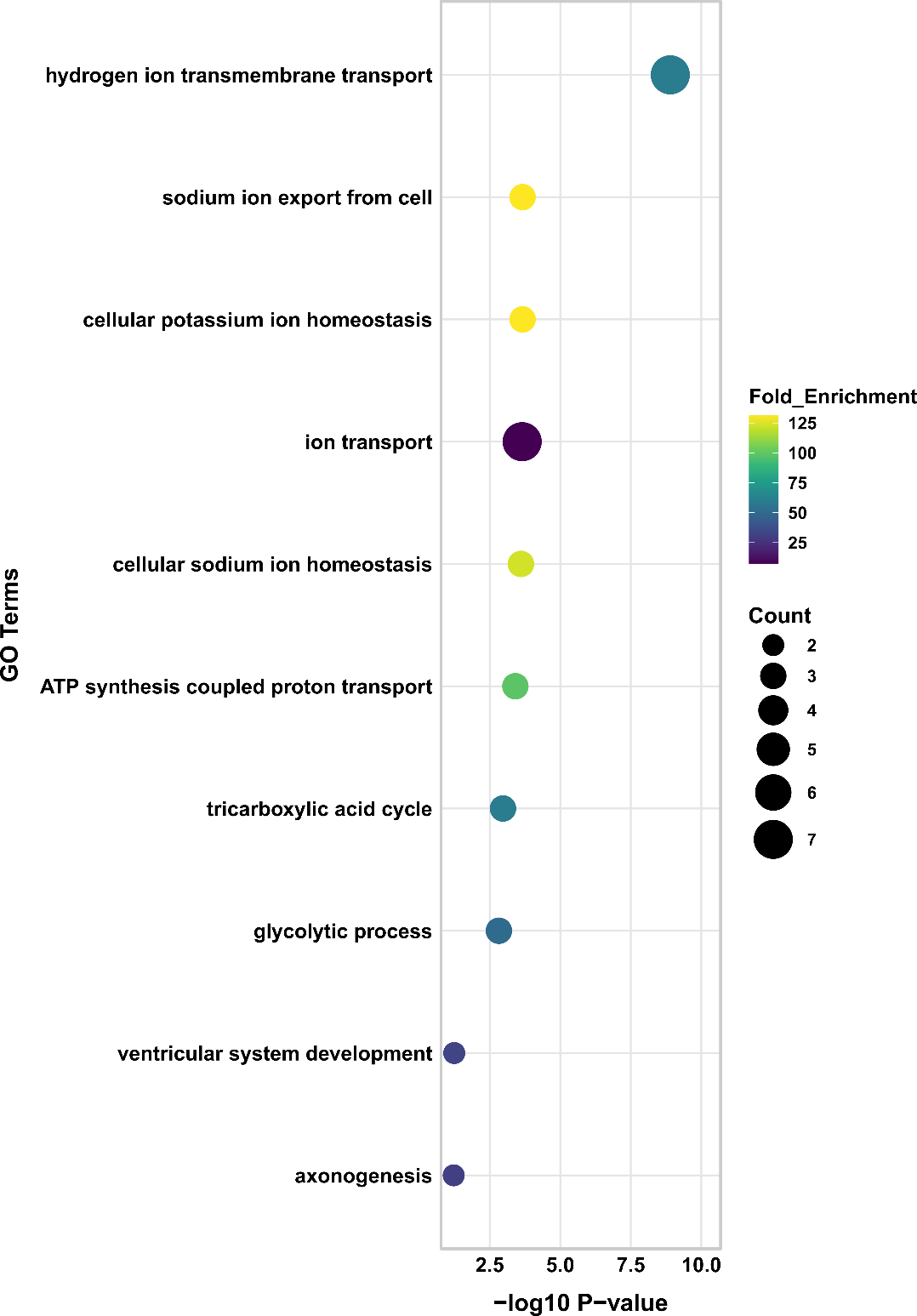
**

**Figure S9**. Gene Ontology (GO) enrichment analysis using DAVID tool [1] of differentially expressed proteoforms in the 16-month-old female and male brains. The X-axis represents the -log₁₀(p-value), indicating the significance of enrichment, while the Y-axis lists the specific Biological Processes. Bubble size corresponds to the number of genes involved, and the color gradient reflects the fold enrichment, with higher values indicating stronger enrichment.


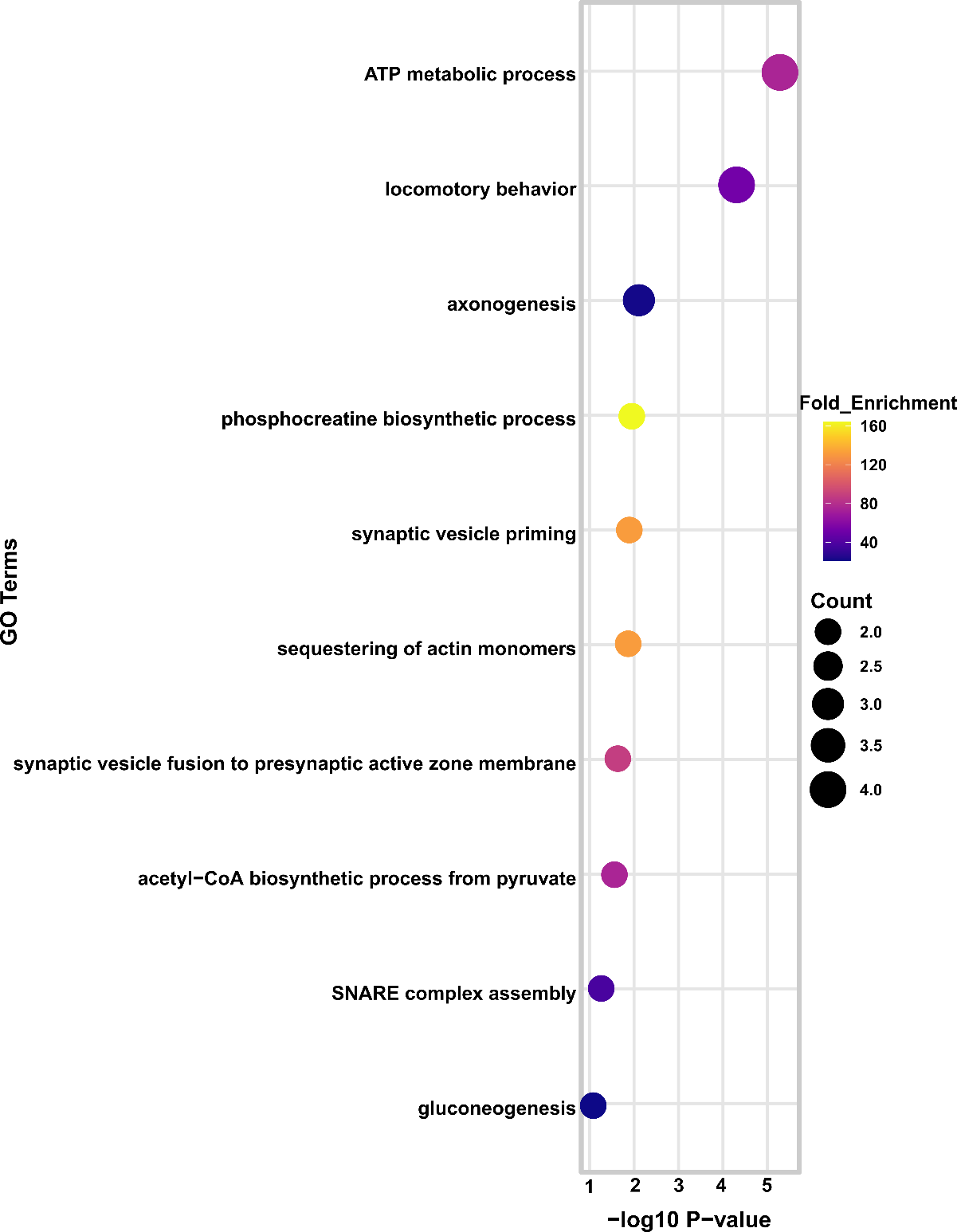


**Figure S10**. Gene Ontology (GO) enrichment analysis using DAVID tool [1] of differentially expressed proteoforms in the 24-month-old female and male brains. The X-axis represents the -log₁₀(p-value), indicating the significance of enrichment, while the Y-axis lists the specific Biological Processes. Bubble size corresponds to the number of genes involved, and the color gradient reflects the fold enrichment, with higher values indicating stronger enrichment.

**Reference:**

1. Sherman, B.T., et al., *DAVID Ortholog: an integrative tool to enhance functional analysis through orthologs.* Bioinformatics, 2024. **40**(10).
